# Deep learning reconstruction for detection of liver lesions at standard-dose and reduced-dose abdominal CT

**DOI:** 10.1007/s00330-025-11596-z

**Published:** 2025-04-19

**Authors:** Tormund H. Njølstad, Kristin Jensen, Hilde K. Andersen, Audun E. Berstad, Gaute Hagen, Cathrine K. Johansen, Kjetil Øye, Jan Glittum, Anniken Dybwad, Emma Thingstad, Marianne G. Guren, Johann Baptist Dormagen, Anselm Schulz

**Affiliations:** 1 Department of Radiology, Haukeland University Hospital, Bergen, Norway; 2 Department of Physics and Computational Radiology, Oslo University Hospital, Oslo, Norway; 3 Division of Radiology and Nuclear Medicine, Oslo University Hospital, Oslo, Norway; 4 Institute of Clinical Medicine, University of Oslo, Oslo, Norway; 5 Department of Oncology, Oslo University Hospital, Oslo, Norway

**Keywords:** Deep learning, Image processing, Computer-assisted, Liver, Multidetector computed tomography

## Abstract

**Objectives:**

Deep learning reconstruction (DLR) has shown promising image denoising ability, but its radiation dose reduction potential remains unknown. The objective of this study was to investigate the diagnostic performance of DLR compared to iterative reconstruction (IR) in the detection of liver lesions at standard-dose and reduced-dose CT.

**Materials and methods:**

Participants with known liver metastases from gastrointestinal and pancreatic adenocarcinoma were prospectively included from routine follow-up (October 2020 to March 2022). Participants received standard-dose CT and two additional reduced-dose scans during the same contrast administration, each reconstructed with IR and high-strength DLR. Two radiologists evaluated images for the presence of liver lesions, and a third established a reference standard. Diagnostic performance was compared using McNemar’s test and mixed effects logistic regression.

**Results:**

Forty-four participants (mean age 66 years ± 11 [standard deviation], 28 men) were evaluated with 348 included liver lesions ≤ 20 mm (297 metastases, 51 benign; mean size 9.1 ± 4.3 mm). Mean volume CT dose index was 14.2, 7.8 mGy, and 5.1 mGy. Between algorithms, no significant difference in lesion detection was observed within dose levels. Detection of 233 lesions ≤ 10 mm was deteriorated with lower dose levels despite DLR denoising, with 185 detected at standard-dose IR (79.4%; 95% CI: 73.5–84.3) vs 128 at medium-dose DLR (54.9%; 95% CI: 48.3–61.4; *p* < 0.001) and 105 at low-dose DLR (45.1%; 95% CI: 38.6–51.7; *p* < 0.001).

**Conclusion:**

Diagnostic performance for liver lesion detection was comparable between algorithms. When the detection of smaller lesions is important, DLR did not facilitate substantial dose reduction.

**Key Points:**

***Question*** *Methods to reduce CT radiation dose are desirable in clinical practice, and DLR has shown promising image denoising capabilities*.

***Findings*** *Liver lesion detection was comparable for DLR and IR across dose levels, but detection of smaller lesions deteriorated with lower dose levels*.

***Clinical relevance*** *Although potent in image noise reduction, the diagnostic performance of DLR is comparable to IR at standard-dose and reduced-dose CT. Care must be taken in pursuit of dose reduction when the detection and characterization of smaller liver lesions are of clinical importance*.

**Graphical Abstract:**

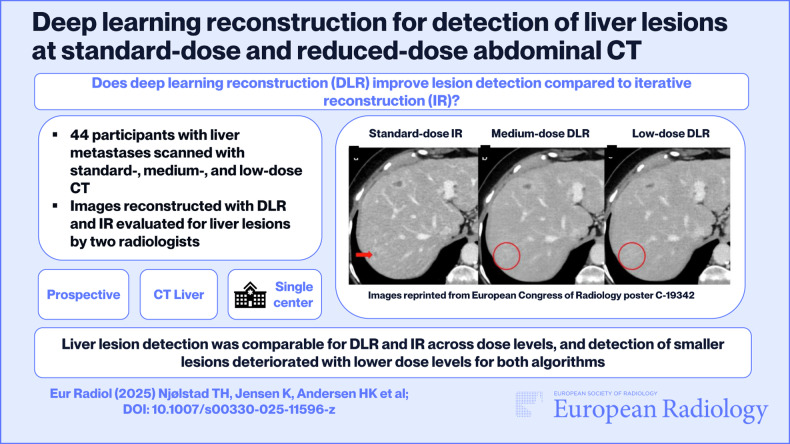

## Introduction

Detection and characterization of liver lesions can be a challenging task in abdominal CT imaging, with considerable impact on clinical care in an oncological setting [1, 2]. With decreasing radiation dose level, such low-contrast tasks become increasingly difficult, primarily attributed to an increase in image noise [3]. Thus, relatively high dose levels may be justified in the appropriate clinical setting to ensure that images are of sufficient diagnostic quality [4]. However, due to long-term radiation-related risks, efforts are made to tailor CT protocols to balance sufficient image quality to identify relevant pathology while keeping radiation dose as low as reasonably achievable (i.e., the ALARA principle) [5–7]. Hence, methods to reduce radiation dose while maintaining acceptable diagnostic image quality are aspired to in clinical practice.

Filtered back projection (FBP) was previously the dominant CT image reconstruction technique primarily due to computational efficiency [8, 9]. Over the last decades, iterative reconstruction (IR) algorithms have been increasingly implemented to reduce image noise [10, 11]. However, although potent in image noise reduction, IR also alters image noise texture, where images have been reported as appearing more ‘blotchy’ or ‘plastic’ compared to FBP and potentially deteriorating low-contrast task performance [12, 13].

Over the last few years, deep learning reconstruction (DLR) has gained traction in clinical practice. Trained with high- and low-dose datasets, these vendor-specific algorithms (such as TrueFidelity [GE Healthcare], AiCE [Canon Medical Systems], and Precise Image [Philips Healthcare]) strive to enhance image quality by reducing image noise in previously challenging areas such as low-dose imaging and scanning of obese individuals [14]. However, although promising, it is imperative that novel reconstruction algorithms undergo rigorous evaluation to ensure that performance is not compromised in pursuit of dose reduction. Phantom studies evaluating the DLR algorithm TrueFidelity (GE Healthcare) have demonstrated robust noise reduction comparable to IR across dose levels without compromising image texture and improved low-contrast detectability [15, 16]. Nevertheless, while clinical studies have shown improved subjective image quality [17, 18], findings are conflicting as to whether or not this translates to substantial dose reduction potential when it comes to low-contrast lesion detection, lesion characterization, and radiologist confidence [19, 20].

On this basis, we set out to investigate the diagnostic performance for low-contrast liver lesion detection using DLR and IR across different dose levels, to assess the potential for dose reduction with DLR in a clinical setting.

## Materials and methods

The study was approved by our regional ethics committee (REK 108784) and data protection officer (PVO 20/18032). Written informed consent was obtained from all participants. Preliminary results based on an initial subset of participants (*n* = 25) have been published as a European Congress of Radiology poster in 2023 [21].

### Study participants

**Fig. 1:**
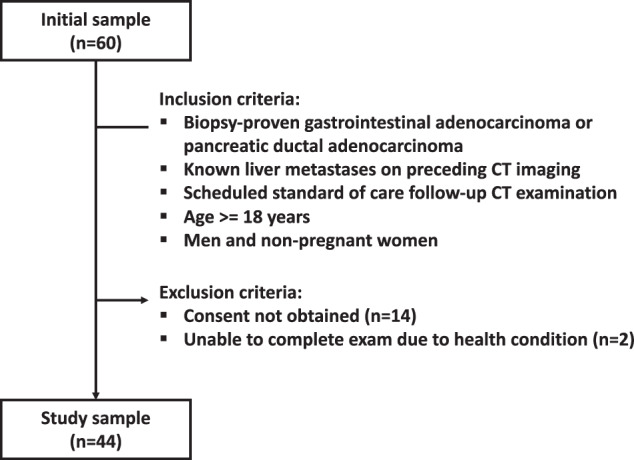
Study flowchart

Power analysis indicated 235 liver lesions needed, assuming a standard dose detection rate of 95% [4, 22], non-inferiority margin of −5%, statistical power of 80%, and one-sided significance level of 5%. Participants with known liver metastasis from gastrointestinal or pancreatic adenocarcinoma were prospectively included from routine follow-up from October 2020 to March 2022, with inclusion and exclusion criteria as shown in Fig. 1. Each participant’s age, sex, height, and weight were recorded. Follow-up of participants to assess the reference standard was conducted until March 2023.

### CT image acquisition and reconstruction

All participants received clinically indicated abdominal CT on a 256-slice CT system (GE Revolution, GE Healthcare), with settings as depicted in Table 1. Contrast material (iohexol 350 mg/mL, Omnipaque, GE Healthcare) was intravenously administered with a weight-based reference of 2 mL per kg, but adjustable to within 1.5–2.5 mL per kg depending on body habitus according to our routine protocol for abdominal CT. Reference contrast material injection time was set to 40 s, and scans were obtained in the portal venous phase, 80 s after contrast material injection. Immediately following the clinically indicated scan and during the same contrast administration, participants received two additional scans covering the upper abdomen with adjusted scanner settings to obtain a medium-dose scan and a low-dose scan. The order of the supplementary scans was altered between participants, and the volume CT dose index (CTDIvol) was recorded for all scans. CT images with 2.5 mm slice thickness were reconstructed using hybrid IR with 50% blend (ASIR-V, GE Healthcare) and high-strength DLR (TrueFidelity, GE Healthcare). A high-strength DLR algorithm was chosen as it provides the highest magnitude of image denoising, with demonstrated improved low-contrast lesion detection in phantoms [15, 16].

**Table 1:** Parameters and data for the abdominal CT protocol

Parameter	Data
Scanner model	GE revolution, 160 mm detector
Detector collimation (mm)	80 (128 × 0.625)
Pitch	0.5
Rotation speed (s)	0.5
Radiation dose modulation	
Standard-dose scan	Standard scanner settings as per institutional protocol for abdominal CT^a,b^
Medium-dose scan	Noise index adjusted to reduce dose to approximately 50–60% of the standard-dose scan
Low-dose scan	Noise index adjusted to reduce dose to approximately 30–40% of the standard-dose scan
Reconstruction thickness (mm)	2.5 mm
Reconstruction algorithm	
IR	ASIR-V 50%
DLR	TrueFidelity, high strength
Reconstruction kernel	Standard

*AP* anteroposterior, *ASIR* adaptive statistical iterative reconstruction, *DLR* deep learning reconstruction, *kVp* kilovolt peak, *LAT* lateral, *IR* iterative reconstruction

^a^ Tube potential was automatically selected based on patient characteristics obtained from the scout image (auto prescription): 100 kVp for AP + LAT diameter ≤ 54 cm (minimum tube current 250 mA), 120 kVp for AP + LAT diameter > 54 cm (minimum tube current 150 mA)

^b^ For standard-dose CT, noise index set to 29 (AP + LAT diameters 55–67 cm) or 32 (AP + LAT diameters above 67 cm) for 120 kV and 25 (AP + LAT diameters below 47 cm) or 27 (AP + LAT diameters 47–54 cm) for 100 kV based on patient characteristics obtained from the scout image

### Lesion detection and reference standard

Two board-certified radiologists (C.K.J. and G.H., with > 20 years of experience in abdominal imaging) reviewed each scan for the presence of liver lesions on a dedicated workstation (Sectra IDS7, Sectra) under standard viewing conditions reflecting a clinical environment. Anonymized images were presented with standard window settings for abdominal CT (window width 350 and window level 30), but freely adjustable to suit reader preference. Readers were blinded to participant characteristics, dose level, and reconstruction algorithm applied, and all scans were presented randomly to minimize recall bias. No preceding or follow-up imaging was available during the reading sessions, and no time limit was imposed for review.

For each scan, liver lesions were marked with corresponding levels of confidence (1, most certainly no lesion; 2, low probability for lesion; 3, intermediate probability for lesion; 4, high probability for lesion; and 5, certain lesion) and characterized (1, definitely benign; 2, probably benign; 3, possibly benign but malignancy not excluded; 4, probably malignant; and 5, definitely malignant).

The reference standard was established by a board-certified radiologist (A.S.) with over ten years of experience in abdominal imaging, using the standard-dose scans and by leveraging all available cross-sectional imaging preceding and following the investigated scans.

### Quantitative analysis

Quantitative image quality was assessed by drawing two-dimensional regions of interest (ROIs) measuring approximately 1 cm^2^ using a dedicated workstation (Sectra IDS 7, Sectra): three in the liver parenchyma and one in paraspinal muscle. For each ROI, the average CT number in Hounsfield Units (HU) and standard deviation were recorded, with the latter considered a measure of image noise. For the ROIs in the liver parenchyma, metrics were averaged to calculate signal-to-noise ratio (SNR) using the formula

$${{{\mathrm{SNR}}}}_{{{\mathrm{liver}}}}=\frac{{{{\mathrm{HU}}}}_{{{\mathrm{liver}}}}}{{{{\mathrm{Noise}}}}_{{{\mathrm{liver}}}}}$$

and contrast-to-noise ratio (CNR) using the formula

$${{\mathrm{CNR}}}=\frac{{{{\mathrm{HU}}}}_{{{\mathrm{liver}}}}-{{{\mathrm{HU}}}}_{{{\mathrm{paraspinal\,muscle}}}}}{{{{\mathrm{Noise}}}}_{{{\mathrm{liver}}}}}$$

### Statistical analysis

CT number, noise, SNR, and CNR were summarized using means ± standard deviation and compared using *t*-statistics. Performance metrics for detection, sensitivity, specificity, and accuracy were assessed for each dose level and reconstruction algorithm, including lesions with a diameter of ≤ 20 mm, and compared using McNemar’s test. For these metrics, lesions detected by at least one reader with a reported confidence scale above two (i.e., intermediate probability for lesion or higher) were considered detected for that dose level. For characterization, lesions were considered correct with the reference standard if characterized with a score of two or less for benign lesions (i.e., probably benign or definitely benign) and three or more for metastases (i.e., possibly benign but malignancy not excluded, probably malignant or definitely malignant) if detected and characterized correctly by at least one reader. Lesions detected by readers but not identified as lesions according to the reference standard were considered false-positives. Detection was assessed using a mixed effects logistic regression model, treating dose level, reconstruction algorithm, and lesion size as fixed effects, and patient and lesion as random effects. For this, dose reduction was included as a continuous variable based on actual dose levels obtained from the scanner for each scan. Reader agreement was evaluated using Cohen’s Kappa and interpreted according to Landis and Koch [23]. All tests were two-sided, and *p*-values ≤ 0.05 were considered statistically significant. Statistical tests were performed using R (v.4.3.3, The R Foundation for Statistical Computing) and Stata SE (v.18.5, StataCorp).

## Results

### Participants and lesion characteristics

A flowchart of participant inclusion is presented in Fig. 1, and participant and lesion characteristics are presented in Table 2. Among the 60 included participants, two were excluded due to inability to perform the exam due to health conditions, and fourteen were excluded because consent could not be obtained. Thus, the final study population was comprised of 44 participants (28 men [64%]) with a mean age ± standard deviation of 66.1 ± 10.9 years (range, 40–86 years) mean weight 76.2 ± 14.3 kg (range, 48–110 kg) and mean BMI of 24.8 ± 3.8 kg/m^2^ (range, 18.5–32.9 kg/m^2^). The reference standard identified 419 liver lesions of which 348 were ≤ 20 mm in largest diameter, 297 (85.3%) malignant and 51 (14.7%) benign. Lesion size was on average 9.1 ± 4.3 mm (range, 3–20 mm). Stratifying by size, 115 lesions (33.0%) were > 10 mm in diameter, 169 lesions (48.6%) were > 5 and ≤ 10 mm in diameter, and 64 lesions (18.4%) were ≤ 5 mm in diameter. Participants had an average of 7.9 lesions and a median of 6 lesions (range, 1–28 lesions).

**Table 2:** Participant demographics and lesion characteristics

Parameter	Value
Participants	44
Male	28 (63.6%)
Female	16 (36.4%)
Age (years)^a^	66.1 ± 10.9
Height (cm)^a^	175 ± 10
Weight (kg)^a^	76.2 ± 14.3
Body mass index (kg/m^2^)^a^	24.8 ± 3.8
Malignancy type	
Colorectal adenocarcinoma	31 (70.5%)
Pancreatic ductal adenocarcinoma	6 (13.6%)
Esophageal adenocarcinoma	7 (15.9%)
Liver lesions^b^	348
Malignant	297 (85.3%)
Benign	51 (14.7%)
Lesion size (mm)^a,b^	9.1 ± 4.3
> 10 mm	115 (33.0%)
> 5 and ≤ 10 mm	169 (48.6%)
≤ 5 mm	64 (18.4%)

^a^ Data are presented as mean ± standard deviation

^b^ Only lesions ≤ 20 mm were included

### Radiation dose and tube potential

Mean CTDI_vol_ ± standard deviation was 14.2 ± 3.6 mGy (range, 9.6–26.3 mGy) for the standard-dose scan, 7.8 ± 2.6 mGy (range, 3.6–17.5 mGy) for the medium-dose scan, and 5.1 ± 2.1 mGy (range, 2.1–11.3 mGy) for the low-dose scan. Correspondingly, with standard-dose CT as reference, the mean dose was 54% ± 8 (range, 35–68%) for the medium-dose scan and 36% ± 7 (range, 21–47%) for the low-dose scan. Thirteen participants (29.5%) were scanned with a tube potential of 100 kV and 31 (70.5%) with a tube potential of 120 kV.

### Contrast material administration

Participants were administered a median intravenous contrast material volume of 150 mL (range, 100–180 mL) with a median flow rate of 3.5 mL/s (range, 2.5–4.0 mL/s).

### Quantitative image quality

For quantitative analyses, ROIs from a total of 264 image sets were included, and results are depicted in Table 3. On average, image noise was 40.3% lower and CNR 67.0% higher with DLR compared to IR (both *p* < 0.001 for difference). There was no significant difference in CT numbers between reconstruction algorithms for each dose level. However, there was a slight decrease in average CT number in the liver parenchyma with lower dose levels, 6.0 HU lower for medium-dose CT and 6.7 HU lower for low-dose CT with standard-dose CT as reference (both *p* < 0.001 for difference). This difference was attributed to a minor difference in liver contrast material enhancement due to a slight scanning delay for the lower dose scans as there was less difference in CT number across dose levels for the ROI placed in paraspinal muscle (0.8 HU lower for medium-dose CT and 0.7 HU lower for low-dose CT with standard-dose CT as reference, *p* = 0.046 and *p* = 0.09 for difference, respectively). Noteworthy, the difference in average CT number in the liver parenchyma between the medium-dose and low-dose scan was 0.7 HU (*p* = 0.051 for difference).

**Table 3:** Quantitative image quality parameters for IR and DLR at standard-dose and reduced-dose CT

	Standard-dose CT		Medium-dose CT		Low-dose CT	
Variable	IR	DLR	IR	DLR	IR	DLR
CT number (HU)						
Liver	128.4 ± 17.7	128.6 ± 17.5	122.4 ± 16.2	122.6 ± 15.6	121.7 ± 16.3	121.9 ± 16.4
Muscle	66.2 ± 8.7	65.6 ± 8.1	66.8 ± 9.2	66.5 ± 8.3	66.2 ± 8.7	66.9 ± 8.1
Noise						
Liver	11.2 ± 1.2	7.0 ± 0.8	13.5 ± 1.3	8.1 ± 0.9	15.4 ± 1.5	8.8 ± 1.0
Muscle	11.1 ± 2.3	7.1 ± 1.1	13.0 ± 2.3	7.9 ± 1.0	15.1 ± 2.4	8.7 ± 1.1
SNR						
Liver	11.6 ± 2.2	18.6 ± 3.6	9.2 ± 1.6	15.3 ± 2.7	8.0 ± 1.3	14.0 ± 2.4
Muscle	6.3 ± 1.8	9.5 ± 2.0	5.3 ± 1.4	8.6 ± 1.8	4.5 ± 1.0	7.8 ± 1.5
CNR						
Liver to muscle	5.6 ± 1.8	9.1 ± 3.0	4.2 ± 1.3	7.0 ± 2.1	3.6 ± 1.1	6.3 ± 1.9

Data are presented as means ± standard deviations

*CNR* contrast-to-noise ratio, *DLR* deep learning reconstruction, *HU* Hounsfield units, *IR* iterative reconstruction, *SNR* signal-to-noise ratio

### Diagnostic accuracy

**Fig. 2:**
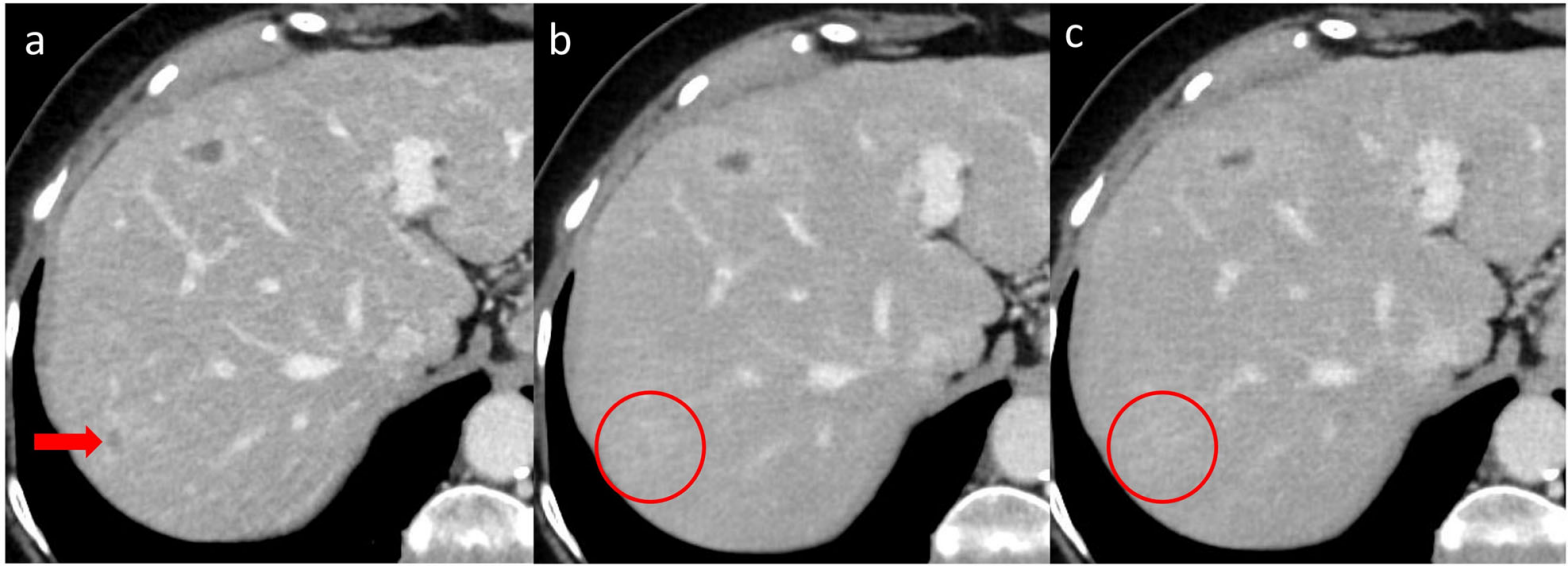
Axial contrast-enhanced CT images obtained with IR at standard-dose CT (**a**), DLR at medium-dose CT (**b**), and DLR at low-dose CT (**c**). A small, 9 mm hypodense liver lesion was detected by both readers at standard-dose IR (red arrow) but missed by both readers at lower dose levels with DLR (red circle). Reprinted, with permission, from reference [21]

For standard-dose CT, reader 1 and reader 2 correctly identified 252 and 247 lesions for IR vs 253 and 251 lesions for DLR, respectively. For medium-dose CT, reader 1 and reader 2 correctly identified 197 and 194 lesions for IR vs 199 and 202 lesions for DLR, respectively. Finally, for low-dose CT, reader 1 and reader 2 correctly identified 175 and 177 lesions for IR vs 177 and 179 lesions for DLR, respectively. The number of false-positive lesions was 25 for IR vs 21 for DLR at standard-dose CT, 15 for IR vs 18 for DLR at medium-dose CT, and 24 for IR vs 20 for DLR at low-dose CT, respectively. These were areas in the liver parenchyma marked by readers considered pseudolesions by the reference standard when leveraging all available cross-sectional imaging, such as intrahepatic vessels, perfusion changes, bile-duct branches, and focal fatty sparing or infiltration. Diagnostic performance metrics for each dose level and subgroup analyses by lesion size are portrayed in Table 4. For standard-dose CT, 83.0% (95% CI: 78.6–86.8%) of lesions were detected for both IR and DLR (*p* > 0.99 for difference). Compared to standard-dose IR, significantly fewer lesions were detected at medium-dose DLR (65.2%; 95% CI: 59.9–70.2%; *p* < 0.001 for difference) and at low-dose DLR (58.9%; 95% CI: 53.5–64.1%; *p* < 0.001 for difference). There was no significant difference in detection between IR and DLR within the same dose level. For lesions larger than 1 cm, there was a slight, but not significant, decline in detection from 90.4% (95% CI: 83.2–94.9%) at standard-dose IR to 86.1% (95% CI: 78.1–91.6%) at medium-dose DLR (*p* = 0.18 for difference) and 87.0% (95% CI: 79.1–92.3%) at low-dose DLR (*p* = 0.34 for difference). For the 233 lesions ≤ 10 mm in size, there was a significant decline in detection from 79.4% (95% CI: 73.5–84.3%) at standard-dose IR to 54.9% (95% CI: 48.3–61.4%) at medium-dose DLR (*p* < 0.001 for difference) and 45.1% (95% CI: 38.6–51.7%) at low-dose DLR (*p* < 0.001 for difference). Correspondingly, when considering only the 192 malignant lesions ≤ 10 mm in size, there was a similar significant deterioration in sensitivity from 79.7% (95% CI: 73.2–85.0%) at standard-dose IR to 50.5% (95% CI: 43.3–57.8%) at medium-dose DLR (*p* < 0.001 for difference) and 43.2% (95% CI: 36.2–50.6%) at low-dose DLR (*p* < 0.001 for difference). Figure 2 demonstrates a 9 mm liver metastasis correctly detected at standard-dose CT with IR but missed by both readers on the medium- and low-dose scans with DLR. Reader agreement was moderate to substantial, with Cohen’s Kappa for IR and DLR estimated to 0.44 and 0.47 for standard-dose CT, 0.69 and 0.69 for medium-dose CT, and 0.71 and 0.69 for low-dose CT, respectively.

**Table 4:** Diagnostic performance for IR and DLR at standard-dose and reduced-dose CT

		Standard-dose CT		Medium-dose CT		Low-dose CT	
Lesion size		IR	DLR	IR	DLR	IR	DLR
All lesions	Detection (%)	83.0 (78.6–86.8) [289/348]	83.0 (78.6–86.8) [289/348]	63.8 (58.5–68.8) [222/348]	65.2 (59.9–70.2) [227/348]	57.8 (52.4–63.0) [201/348]	58.9 (53.5–64.1) [205/348]
	Sensitivity (%)	83.8 (79.0–87.7) [249/297]	85.2 (80.5–88.9) [253/297]	62.6 (56.8–68.1) [186/297]	63.3 (57.5–68.7) [188/297]	55.9 (50.0–61.6) [166/297]	58.2 (52.4–63.9) [173/297]
	Specificity (%)	33.3 (22.1–48.0) [17/51]	17.6 (8.9–31.4) [9/51]	19.6 (10.3–33.5) 10/51	17.6 (8.9–31.4) [9/51]	27.5 (16.3–42) 14/51	23.5 (13.2–37.8) 12/51
	Accuracy (%)	76.4 (71.6–80.7) 266/348	75.3 (70.3–79.7) 262/348	56.3 (50.9–61.6) 196/348	56.6 (51.2–61.9) 197/348	51.7 (46.3–57.1) 180/348	53.2 (47.8–58.5) 185/348
> 10 mm	Detection (%)	90.4 (83.2–94.9) [104/115]	91.3 (84.2–95.5) [105/115]	80.0 (71.3–86.7) [92/115]	86.1 (78.1–91.6) [99/115]	86.1 (78.1–91.6) [99/115]	87.0 (79.1–92.3) [100/115]
	Sensitivity (%)	91.4 (83.9–95.8) [96/105]	92.4 (85.1–96.4) [97/105]	80.0 (70.8–86.9) [84/105]	86.7 (78.3–92.3) [91/105]	84.8 (76.1–90.8) [89/105]	85.7 (77.2–91.5) [90/105]
	Specificity (%)	30.0 (8.1–64.6) [3/10]	20.0 (3.5–55.8) [2/10]	20.0 (3.5–55.8) [2/10]	20.0 (3.5–55.8) [2/10]	30.0 (8.1–64.6) [3/10]	30.0 (8.1–64.6) [3/10]
	Accuracy (%)	86.1 (78.1–91.6) [99/115]	86.1 (78.1–91.6) [99/115]	74.8 (65.7–82.2) [86/115]	80.9 (72.3–87.4) [93/115]	80.0 (71.3–86.7) [92/115]	80.9 (72.3–87.4) [93/115]
> 5 to ≤ 10 mm	Detection (%)	84.0 (77.4–89.0) [142/169]	84.0 (77.4–89.0) [142/169]	58.0 (50.2–65.5) [98/169]	58.6 (50.7–66.0) [99/169]	47.9 (40.2–55.7) [81/169]	52.7 (44.9–60.3) [89/169]
	Sensitivity (%)	84.4 (77.5–89.6) [130/154]	84.4 (77.5–89.6) [130/154]	56.5 (48.3–64.4) [87/154]	55.8 (47.6–63.8) [86/154]	45.5 (37.5–53.7) [70/154]	50.6 (42.5–58.7) [78/154]
	Specificity (%)	20.0 (5.3–48.6) [3/15]	6.7 (0.3–34.0) [1/15]	6.7 (0.3–34.0) [1/15]	13.3 (2.3–41.6) [2/15]	20.0 (5.3–48.6) [3/15]	20.0 (5.3–48.6) [3/15]
	Accuracy (%)	78.7 (71.6–84.5) [133/169]	77.5 (70.3–83.4) [131/169]	52.1 (44.3–59.8) [88/169]	52.1 (44.3–59.8) [88/169]	43.2 (35.7–51.0) [73/169]	47.9 (40.2–55.7) [81/169]
≤ 5 mm	Detection (%)	67.2 (54.2–78.1) [43/64]	65.6 (52.6–76.8) [42/64]	50.0 (38.1–61.9) [32/64]	45.3 (33.0–58.2) [29/64]	32.8 (21.9–45.8) [21/64]	25.0 (15.4–37.7) [16/64]
	Sensitivity (%)	60.5 (43.5–75.5) [23/38]	68.4 (51.2–82.0) [26/38]	39.5 (24.5–56.5) [15/38]	28.9 (16.0–46.1) [11/38]	18.4 (8.3–34.9) [7/38]	13.2 (4.9–28.9) [5/38]
	Specificity (%)	42.3 (24.0–62.8) [11/26]	23.1 (9.8–44.1) [6/26]	26.9 (12.4–48.1) [7/26]	19.2 (7.3–40.0) [5/26]	30.7 (15.1–51.9) [8/26]	23.1 (9.8–44.1) [6/26]
	Accuracy (%)	53.1 (40.3–65.5) [34/64]	50.0 (38.1–61.9) [32/64]	34.4 (23.2–47.4) [22/64]	25.0 (15.4–37.7) [16/64]	23.4 (14.1–36.0) [15/64]	17.2 (9.3–29.1) [11/64]

95% confidence intervals in parentheses; number of lesions in brackets

*DLR* deep learning reconstruction, *IR* iterative reconstruction

**Fig. 3:**
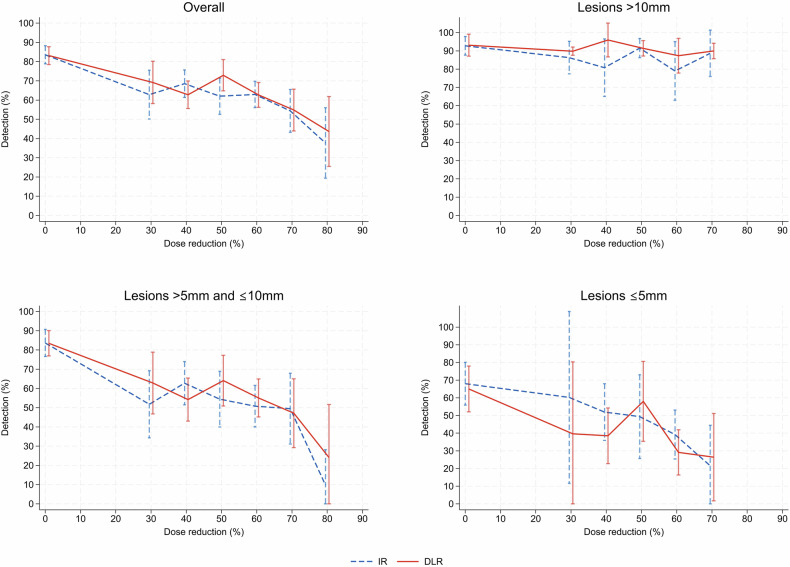
Results from mixed effects logistic regression, with line graphs of estimated liver lesion detection by reduction in radiation dose from full dose level obtained from scanner grouped to nearest ten percent for IR and DLR for all lesions and stratified by lesion size. Error bars represent 95% confidence intervals

Mixed-effects logistic regression for detection with dose level, reconstruction algorithm, and lesion size as fixed effects and patient and lesion as random effects showed no significant difference in detection between reconstruction algorithms (estimated OR 1.11 for DLR compared to IR; 95% CI: 0.82–1.49, *p* = 0.68) and a significant deterioration in detection with reduced dose level (estimated OR 0.95 for lesion detection associated with a decrease in dose level by one percentage point; 95% CI: 0.95–0.96, *p* < 0.001). An overview of estimated detection by dose reduction for each reconstruction algorithm is shown in Fig. 3.

## Discussion

Methods to reduce CT image noise in pursuit of dose reduction are desirable in clinical practice. Our study evaluated the diagnostic performance of a high-strength DLR algorithm compared to IR at standard- and reduced-dose CT, and found no significant difference in liver lesion detection. Furthermore, despite DLR denoising, detection of ≤ 10 mm lesions significantly deteriorated with lower dose levels, from 79.4% at standard-dose IR to 54.9% at medium-dose DLR and 45.1% at low-dose DLR.

Several studies have demonstrated improved image quality with DLR. Phantom studies have shown robust image denoising at comparable levels to higher blends of hybrid IR, while preserving noise texture similar to FBP [24, 25]. Semi-anthropomorphic phantom studies have shown improved low-contrast lesion detection, with increased performance with higher DLR strength [16, 26]. In a clinical setting, DLR has shown improved perceived overall image quality and lesion diagnostic confidence. Nonetheless, some readers have noted a minor blurring of < 5 mm liver lesions with higher DLR strengths [18]. The important question is whether this improved subjective image quality translates to improved diagnostic performance or significant dose reduction.

Interestingly, our study demonstrates that diagnostic performance for liver lesion detection is not improved, and deteriorates for smaller liver lesions when the dose level is reduced, despite DLR image denoising; thereby limiting dose reduction potential when detection of smaller liver lesions is of importance. This is in line with a recent study by Jensen et al [19], demonstrating a decline in detection of 65 small liver lesions (≤ 5 mm) from 96.9% with standard-dose FBP to 72.3% with reduced-dose medium-strength DLR. Noticeably, relatively high dose levels were applied, with reported mean CTDIvol of 34.9 mGy for full-dose CT and 12.2 mGy for reduced-dose CT. Thus, our study is an important supplement as we explore performance using a higher strength of DLR with more rigorous image denoising, across lower dose levels (mean CTDIvol of 14.2 mGy, 7.8 mGy, and 5.1 mGy), and evaluate both DLR and IR for each investigated dose level. Noticeably, Caruso et al [20] found a slight difference at standard-dose CT (mean CTDIvol 24.1 mGy) in the detection of 33 liver lesions ≤ 5 mm (32 with medium-strength DLR vs 26 for IR; *p* = 0.031 for difference), concluding that DLR may allow for dose reduction, subject to validation in larger studies. However, Cao et al [27] retrospectively found no difference between IR and DLR of low, medium, and high strength in the detection of 25 lesions ≤ 10 mm at standard-dose CT. Our study thus contributes to existing literature, leveraging a large sample size of 348 liver lesions, with 233 small (≤ 10 mm) and 64 of these very small (≤ 5 mm), and indicating that rigorous denoising does not translate to improved lesion detection in practice.

As detection of lesions > 10 mm was not significantly deteriorated at lower dose levels, there may be a dose reduction potential when detection and characterization of smaller liver lesions are of less importance. Arguably, care must be taken to tailor CT protocols in balancing image quality with radiation-related risks, where low-dose imaging may be justified in the appropriate clinical setting, such as pediatric imaging, multiple contrast phase scans, and frequent follow-up examinations [28]. For this, prospective clinical trials in both pediatric and adult settings assessing a broader spectrum of oncological and non-oncological pathologies, preferably exploring different contrast phases and DLR algorithms of different strengths and from different vendors, are needed to fully characterize the dose reduction potential using DLR.

Our study is not without limitations. First, we explored one vendor-specific DLR algorithm assessing a liver low-contrast task, and findings are not necessarily transferable to other vendor-specific algorithms and different pathologies in different organs. Secondly, we only compared one strength of DLR and a blend of IR, where ideally a broader spectrum of algorithms could have been assessed. However, the increasing number of investigated algorithms runs the risk of creating overly strenuous reader sessions. For IR, a 50% blend was applied, reflecting our clinical routine for abdominal CT, whereas a high-strength DLR algorithm was chosen as it provides the highest magnitude of image denoising, with demonstrated improved low-contrast lesion detection in phantoms [16, 17, 25]. Third, as we deliberately restricted the study population to histologically confirmed metastatic disease from gastrointestinal cancer, our findings may not necessarily reflect performance in scenarios involving a range of other hepatic lesions in a non-oncologic setting. Finally, we observed a slight reduction in mean CT number in the liver parenchyma with the reduced-dose scans, attributed to a slight scanning delay, and cannot exclude that this impacted detection performance at lower dose levels. However, comparing algorithms within the same dose level arguably mitigates this limitation. Furthermore, given the randomized order for the two low-dose scans, the difference in mean CT-number between the two low-dose scans was only 0.7 HU. As we consistently observed a decline in lesion conspicuity for the lowest dose level, which was also evident in the linear mixed regression model, we perceive radiation dose to be the primary driver of reduced performance with lower dose level, and not the slight time-dependent reduction in contrast although we cannot exclude that this may have been a contributing factor when comparing the two lowest dose levels to the standard-dose scan.

In conclusion, DLR improved image noise and contrast-to-noise ratio relative to IR for standard-dose and reduced-dose abdominal CT, but did not improve liver lesion detection. Furthermore, detection of liver lesions ≤ 10 mm was significantly reduced with decreasing dose level for both algorithms. Thus, care must be taken in pursuit of dose reduction when the detection of smaller liver lesions is of importance. As detection did not significantly deteriorate for lesions > 10 mm, there may be potential for dose reduction in the appropriate clinical setting, such as follow-up scenarios for stable diseases and in a non-oncological or screening setting where detection of larger lesions is sufficient, subject to clinical validation.

## Abbreviations

CTDIvol: Volume CT dose index
DLR: Deep learning reconstruction
FBP: Filtered back projection
IR: Iterative reconstruction

## References

[1] Fletcher JG, Yu L, Li Z et al (2015) Observer performance in the detection and classification of malignant hepatic nodules and masses with CT image-space denoising and iterative reconstruction. Radiology 276:465–478 26020436 10.1148/radiol.2015141991 PMC4514571

[2] Morris VK, Kennedy EB, Baxter NN et al (2023) Treatment of metastatic colorectal cancer: ASCO guideline. J Clin Oncol 41:678–700 36252154 10.1200/JCO.22.01690 PMC10506310

[3] Solomon J, Marin D, Roy Choudhury K, Patel B, Samei E (2017) Effect of radiation dose reduction and reconstruction algorithm on image noise, contrast, resolution, and detectability of subtle hypoattenuating liver lesions at multidetector CT: filtered back projection versus a commercial model-based iterative reconstruction algorithm. Radiology 284:777–787 28170300 10.1148/radiol.2017161736 PMC5702911

[4] Jensen CT, Wagner-Bartak NA, Vu LN et al (2019) Detection of colorectal hepatic metastases is superior at standard radiation dose CT versus reduced dose CT. Radiology 290:400–409 30480489 10.1148/radiol.2018181657 PMC6357984

[5] Guideline (1991) 1990 Recommendations of the International Commission on Radiological Protection. Ann ICRP 21:1–201 2053748

[6] Smith-Bindman R, Lipson J, Marcus R et al (2009) Radiation dose associated with common computed tomography examinations and the associated lifetime attributable risk of cancer. Arch Int Med 169:2078–2086 20008690 10.1001/archinternmed.2009.427 PMC4635397

[7] Bosch de Basea Gomez M, Thierry-Chef I, Harbron R et al (2023) Risk of hematological malignancies from CT radiation exposure in children, adolescents and young adults. Nat Med 29:3111–3119 37946058 10.1038/s41591-023-02620-0 PMC10719096

[8] Willemink MJ, Noel PB (2019) The evolution of image reconstruction for CT-from filtered back projection to artificial intelligence. Eur Radiol 29:2185–2195 30377791 10.1007/s00330-018-5810-7 PMC6443602

[9] Schofield R, King L, Tayal U et al (2020) Image reconstruction: part 1–understanding filtered back projection, noise and image acquisition. J Cardiovasc Comput Tomogr 14:219–225 31023632 10.1016/j.jcct.2019.04.008

[10] Geyer LL, Schoepf UJ, Meinel FG et al (2015) State of the art: iterative CT reconstruction techniques. Radiology 276:339–357 26203706 10.1148/radiol.2015132766

[11] Willemink MJ, de Jong PA, Leiner T et al (2013) Iterative reconstruction techniques for computed tomography Part 1: technical principles. Eur Radiol 23:1623–1631 23314600 10.1007/s00330-012-2765-y

[12] Singh S, Kalra MK, Hsieh J et al (2010) Abdominal CT: comparison of adaptive statistical iterative and filtered back projection reconstruction techniques. Radiology 257:373–383 20829535 10.1148/radiol.10092212

[13] Mileto A, Guimaraes LS, McCollough CH, Fletcher JG, Yu L (2019) State of the art in abdominal CT: the limits of iterative reconstruction algorithms. Radiology 293:491–503 31660806 10.1148/radiol.2019191422

[14] Koetzier LR, Mastrodicasa D, Szczykutowicz TP et al (2023) Deep learning image reconstruction for CT: technical principles and clinical prospects. Radiology 306:e221257 36719287 10.1148/radiol.221257 PMC9968777

[15] Greffier J, Hamard A, Pereira F et al (2020) Image quality and dose reduction opportunity of deep learning image reconstruction algorithm for CT: a phantom study. Eur Radiol 30:3951–3959 32100091 10.1007/s00330-020-06724-w

[16] Njølstad T, Jensen K, Dybwad A, Salvesen Ø, Andersen HK, Schulz A (2022) Low-contrast detectability and potential for radiation dose reduction using deep learning image reconstruction—a 20-reader study on a semi-anthropomorphic liver phantom. Eur J Radiol Open 9:100418 35391822 10.1016/j.ejro.2022.100418 PMC8980706

[17] Njølstad T, Schulz A, Godt JC et al (2021) Improved image quality in abdominal computed tomography reconstructed with a novel Deep Learning Image Reconstruction technique–initial clinical experience. Acta Radiologica Open 10:20584601211008391 33889427 10.1177/20584601211008391 PMC8040588

[18] Jensen CT, Liu X, Tamm EP et al (2020) Image quality assessment of abdominal CT by use of new deep learning image reconstruction: initial experience. AJR Am J Roentgenol 215:50–57 32286872 10.2214/AJR.19.22332

[19] Jensen CT, Gupta S, Saleh MM et al (2022) Reduced-dose deep learning reconstruction for abdominal CT of liver metastases. Radiology 303:90–98 35014900 10.1148/radiol.211838 PMC8962777

[20] Caruso D, De Santis D, Del Gaudio A et al (2024) Low-dose liver CT: image quality and diagnostic accuracy of deep learning image reconstruction algorithm. Eur Radiol 34:2384–2393 37688618 10.1007/s00330-023-10171-8 PMC10957592

[21] Njølstad T, Jensen K, Andersen HK et al (2023) Diagnostic performance of deep learning compared to iterative image reconstruction for detection of liver metastases in reduced-dose abdominal CT. ECR 2023, Poster number C-19342, ESR. 10.26044/ecr2023/C-19342

[22] Fält T, Söderberg M, Hörberg L et al (2019) Simulated dose reduction for abdominal CT with filtered back projection technique: effect on liver lesion detection and characterization. AJR Am J Roentgenol 212:84–93 30299999 10.2214/AJR.17.19441

[23] Landis JR, Koch GG (1977) The measurement of observer agreement for categorical data. Biometrics 33:159–174 843571

[24] Solomon J, Lyu P, Marin D, Samei E (2020) Noise and spatial resolution properties of a commercially available deep-learning based CT reconstruction algorithm. Med Phys 47:3961–3971 32506661 10.1002/mp.14319

[25] Njølstad T, Schulz A, Jensen K, Andersen HK, Martinsen ACT (2023) Improved image quality with deep learning reconstruction—a study on a semi-anthropomorphic upper-abdomen phantom. Res Diagn Interv Imaging 5:100022 39076164 10.1016/j.redii.2023.100022 PMC11265485

[26] Toia GV, Zamora DA, Singleton M et al (2023) Detectability of small low-attenuation lesions with deep learning CT image reconstruction: a 24-reader phantom study. AJR Am J Roentgenol 220:283–295 36129222 10.2214/AJR.22.28407

[27] Cao J, Mroueh N, Mercaldo N et al (2024) Detectability of hypoattenuating liver lesions with deep learning CT reconstruction: a phantom and patient study. Radiology 313:e232749 39377679 10.1148/radiol.232749 PMC11535864

[28] Strauss KJ, Goske MJ, Kaste SC et al (2010) Image gently: ten steps you can take to optimize image quality and lower CT dose for pediatric patients. AJR Am J Roentgenol 194:868–873 20308484 10.2214/AJR.09.4091

